# Regional brain glucose uptake following gastric bypass surgery during normo- and hypoglycemic clamp: a pilot FDG-PET study

**DOI:** 10.1007/s12020-024-04127-1

**Published:** 2024-12-07

**Authors:** Giovanni Fanni, Sofia Kvernby, Sadiq Radhi, Argyri Mathioudaki, Magnus Sundbom, Sven Haller, Erika Roman, Johan Wikström, Mark Lubberink, Jan W. Eriksson

**Affiliations:** 1 Department of Medical Sciences, Clinical Diabetes and Metabolism, Uppsala University, Uppsala, Sweden; 2 Department of Surgical Sciences, Molecular Imaging and Medical Physics, Uppsala University, Uppsala, Sweden; 3 Department of Surgical Sciences, Surgery, Uppsala University, Uppsala, Sweden; 4 Department of Surgical Sciences, Neuroradiology, Uppsala University, Uppsala, Sweden; 5 CIMC—Centre d’Imagerie Médicale de Cornavin, Genève, Switzerland; 6 Department of Pharmaceutical Biosciences, Uppsala University, Uppsala, Sweden; 7 Department of Animal Biosciences, Swedish University of Agricultural Sciences, Uppsala, Sweden

**Keywords:** RYGB, FDG-PET, Brain metabolism, Counterregulatory response.

## Abstract

**Purpose:**

We aimed to characterize the RYGB-induced changes in the dynamics of brain glucose uptake. We addressed heterogeneity between brain regions during experimental normo- and hypoglycemia and explored associations with anthropometric and metabolic outcomes of RYGB.

**Methods:**

Analyses of regional brain glucose uptake were performed on 9 individuals with obesity and no diabetes, investigated with combined brain ^18^F-FDG-PET and fMRI during hyperinsulinemic normo- and hypoglycemic clamp, one month before and four months after RYGB. FDG clearance, reflecting glucose uptake rate, was assessed in 38 brain regions, covering all cortical areas and subcortical nuclei, during hyperinsulinemic normo- and hypoglycemia. Correlation analyses were performed to identify associations with other outcomes of RYGB.

**Results:**

FDG uptake rate during hypoglycemia was higher than during normoglycemia in all brain regions, both before and after RYGB. Moreover, in most regions and especially in cortical areas involved in inhibitory behavioral control, FDG uptake rate tended to be reduced after surgery during normoglycemia but elevated during hypoglycemia. However, these post-surgical changes in FDG uptake rate were opposite in the hypothalamus. Thus, the hypo-to-normoglycemia FDG clearance ratio tended to increase in all brain regions following RYGB, but not in the amygdala and the hypothalamus. Changes in regional FDG uptake rate after RYGB during normoglycemia were associated with weight loss and improved systemic insulin sensitivity.

**Conclusion:**

Using dynamic FDG-PET, we show region-specific patterns of changes in glucose utilization following RYGB. In the hypothalamus, glucose uptake during normoglycemia tended to rise after RYGB while it was reduced in cortical regions involved in behavioral control. Following RYGB, the hypothalamus and amygdala, in contrast to other regions, displayed trends of reduced glucose uptake during hypoglycemia. These pilot results highlight the brain effects of RYGB and suggest behavioral and neuroendocrine adaptations which contribute to its antidiabetic effects.

## Introduction

Roux-en-Y gastric bypass (RYGB) is a well-established surgical procedure performed to treat severe obesity. It exerts beneficial metabolic effects including prevention and even reversal of type 2 diabetes [1]. Its effects are indeed partly mediated by weight loss, but other mechanisms independent of caloric restriction and weight loss are also involved [2, 3], including neuroendocrine adaptations involving the brain and neuroendocrine axes [4, 5]. These adaptations can even lead to postprandial hypoglycemia and long-term hypoglycemia unawareness, suggesting altered glucose sensing in the brain [6]. Recently, an extensive review on the effects of weight loss interventions on brain networks and metabolism has been published [7].

We have recently shown that patients with obesity display changes in brain glucose uptake and cerebral blood flow 4 months after undergoing RYGB, and that this is related to improved cognitive performance under experimental hypoglycemia [8]. Moreover, in the same cohort we showed changes in cortical and subcortical brain functional connectivity during experimental glucose deprivation. This affected regions that are involved in neural pathways responsible for reward, inhibitory behavioral control, and energy homeostasis [9], and these changes were associated with dampened hypothalamus-pituitary-adrenal (HPA)-axis activation in response to hypoglycemia, suggesting their role in mediating the favorable glucometabolic outcome of RYGB [9].

The hypothalamus is the brain’s control unit for energy and glucose homeostasis [10, 11]. Other brain regions and neural circuits, such as the default mode network and the reward system, are also known to play a role in eating behavior and energy homeostasis and are known to be dysregulated in obesity [12, 13]. Since brain activity and neuronal glucose metabolism are assumed to be directly linked [14], an altered neural network activity can be reflected in a change of glucose uptake rate in its anatomical correlates.

In contrast to peripheral insulin-sensitive tissues, glucose uptake in the brain is elevated in insulin-resistant compared to insulin-sensitive individuals when measured during a hyperinsulinemic normoglycemic clamp [15–17]. RYGB can normalize whole-brain glucose uptake rate, and this was shown during both experimental hyperinsulinemic normoglycemia [8, 18] and the oral glucose tolerance test (OGTT) [19]. Increased brain glucose uptake has been associated with abnormal beta-cell function [20], higher plasma glucose [21], endogenous glucose production [5], and inversely with the M-value, a gold standard marker of insulin sensitivity [15, 16]. The latter association was seen subjects with obesity and persisted even after RYGB, whereas it was absent in non-diabetic controls [5].

So far, very few studies highlighted RYGB-associated changes in brain glucose uptake at a regional level. Hunt et al. showed prominent differences in regional brain glucose uptake after RYGB in areas involved in energy homeostasis, hedonic responses, and inhibitory control in response to a standardized meal [22], whilst Dardano et al. illustrated the changes in regional brain glucose metabolic rate during an oral glucose tolerance test [19]. Regional brain glucose uptake in brain lobes and in the midbrain was also measured before and after obesity in a study by Rebelos et al., but no post-surgical comparisons were made [5]. No data are presently available about the RYGB-associated changes in regional brain glucose uptake during hypoglycemia.

The aim of the study was to describe RYGB-induced changes in regional brain glucose uptake across cortical and subcortical regions during hyperinsulinemic normoglycemia and hypoglycemia in individuals with obesity. We hypothesize that regions involved in energy and glucose homeostasis, reward, and behavioral control show altered patterns of glucose uptake rate after RYGB. Therefore, in this post-hoc analysis of our previously published study [8], we characterize the RYGB-associated changes in regional brain glucose metabolism in both the normoglycemic and the hypoglycemic phase of a hyperinsulinemic clamp, and we explore its association with anthropometric, glucometabolic, and cognitive outcomes of the surgical intervention.

## Methods

### Study design and subjects

This work presents post-hoc subanalyses carried out on data from a previously published study [8]. Briefly, the study was carried out on 11 individuals with obesity without diabetes, aged 25–49 and with body mass index (BMI) 35.2–45.4 kg/m^2^, recruited at the obesity outpatient facility at Uppsala University Hospital during a routine visit before a planned RYGB operation. Details of this cohort and study procedures can be found elsewhere [8]. The subjects were investigated around one month before RYGB and 4 months after the intervention. In accordance with local guidelines, the patients underwent a 4-week low-calorie diet (800–1100 kcal/day) before RYGB. The study visits were performed at the PET/MR facility at Uppsala University Hospital and started at 8 am when the subjects were required to show up after an overnight fast. Medical history, anthropometric measures including total body fat assessed with bioimpedance, and blood samples were obtained.

### Investigations

At each visit, the subjects underwent a combined metabolic and neuroimaging assessment with simultaneous PET/MRI imaging and a two-step hyperinsulinemic clamp. This consisted of a first normoglycemic phase (5 mM) and a second hypoglycemic phase (2.7 mM). Whole body insulin sensitivity was assessed with the M-value, calculated as the glucose infusion rate during steady state (last 40 min of the hyperinsulinemic normoglycemic clamp) divided by the participant’s lean body mass [8]. In this phase, plasma glucose was stable and urinary glucose excretion was negligible since study participants did not have diabetes [23].

Cognitive assessments were performed during both the normoglycemic and the hypoglycemic phase using the Trail Making Test (TMT) and the Digital Symbol Substitution Test (DSST). Hypoglycemic symptoms were evaluated during hypoglycemia via the Edinburgh hypoglycemia symptoms score (EHSS). Blood samples were drawn from an antecubital vein after arterialization and were analyzed at the Department of Clinical Chemistry and the Clinical Diabetes Research Laboratory of Uppsala University Hospital. Neuroimaging was performed using an integrated PET/MR scanner (SIGNA; GE Healthcare, Waukesha, WI), comprising a 3 T MRI scanner [8]. Details describing the steps of the whole combined procedure were previously reported [8].

### Brain FDG uptake assessment

One patient was excluded because of missing neuroimaging data and one patient was excluded from the analyses because of data file corruption.

Data of FDG net uptake rate were obtained from 38 regions covering all cortical areas (excluded the cerebellum) and subcortical nuclei (the complete list of available regions is given in Table 1). Brain regions were clustered into functional networks according to an anatomical criterion as follows: (1) the reward network: nucleus accumbens, striatum, superior frontal gyrus, anterior part of cingulate gyrus, insula, hippocampus, hypothalamus, thalamus, pallidum, amygdala); (2) the behavioral control network: middle frontal gyrus, anterior part of cingulate gyrus, and orbitofrontal gyri; (3) the default mode network: superior frontal gyrus, anterior part of cingulate gyrus, posterior part of cingulate gyrus, inferior remainder of the parietal lobe.

**Table 1:** Regional net uptake rates of ^18^F-FDG (Ki) for each analyzed region. Data are presented as mean (SD)

	Before RYGB		After RYGB	
	Normoglycemia	Hypoglycemia	Normoglycemia	Hypoglycemia
Frontal (cortex)	0.019 (0.002)	0.044 (0.013)	0.018 (0.002)	0.044 (0.017)
Gyrus rectus	0.017 (0.002)	0.034 (0.023)	0.017 (0.002)	0.043 (0.016)
Inferior frontal gyrus	0.020 (0.002)	0.049 (0.012)	0.019 (0.002)	0.050 (0.021)
Middle frontal gyrus	0.020 (0.002)	0.047 (0.017)	0.018 (0.002)	0.046 (0.018)
Orbitofrontal gyri	0.018 (0.002)	0.042 (0.016)	0.018 (0.002)	0.048 (0.017)
Precentral gyrus	0.018 (0.002)	0.040 (0.014)	0.017 (0.002)	0.039 (0.018)
Superior frontal gyrus	0.018 (0.002)	0.040 (0.014)	0.017 (0.002)	0.040 (0.016)
Inferolateral remainder of parietal lobe	0.020 (0.002)	0.043 (0.018)	0.018 (0.002)	0.044 (0.018)
Parietal (cortex)	0.019 (0.002)	0.042 (0.017)	0.018 (0.002)	0.043 (0.017)
Postcentral gyrus	0.018 (0.002)	0.038 (0.012)	0.017 (0.002)	0.038 (0.016)
Superior parietal gyrus	0.019 (0.002)	0.044 (0.016)	0.018 (0.002)	0.043 (0.020)
Anterior temporal lobe lateral part	0.013 (0.002)	0.035 (0.013)	0.013 (0.002)	0.031 (0.013)
Anterior temporal lobe medial part	0.011 (0.002)	0.020 (0.015)	0.012 (0.001)	0.022 (0.012)
Fusiform gyrus	0.014 (0.001)	0.028 (0.013)	0.014 (0.002)	0.032 (0.015)
Insula (cortex)	0.018 (0.002)	0.035 (0.017)	0.017 (0.002)	0.040 (0.017)
Med temp lobe	0.012 (0.001)	0.023 (0.014)	0.012 (0.002)	0.023 (0.011)
Middle and inferior temporal gyri	0.017 (0.002)	0.040 (0.014)	0.016 (0.003)	0.043 (0.017)
Posterior temporal lobe	0.017 (0.002)	0.036 (0.015)	0.017 (0.003)	0.041 (0.016)
Superior temporal gyrus	0.019 (0.002)	0.038 (0.017)	0.018 (0.003)	0.042 (0.018)
Temporal (cortex)	0.016 (0.002)	0.034 (0.014)	0.016 (0.002)	0.038 (0.015)
Cuneus	0.021 (0.003)	0.038 (0.021)	0.020 (0.003)	0.045 (0.022)
Lateral remainder of occipital lobe	0.020 (0.002)	0.039 (0.017)	0.018 (0.003)	0.046 (0.020)
Lingual gyrus	0.021 (0.003)	0.040 (0.022)	0.019 (0.002)	0.044 (0.021)
Occipital (cortex)	0.020 (0.002)	0.039 (0.018)	0.018 (0.003)	0.046 (0.020)
Cingulate	0.020 (0.002)	0.050 (0.013)	0.020 (0.002)	0.049 (0.021)
Anterior part of cingulate gyrus	0.019 (0.002)	0.047 (0.012)	0.018 (0.002)	0.047 (0.026)
Posterior part of cingulate gyrus	0.023 (0.003)	0.052 (0.017)	0.021 (0.003)	0.059 (0.024)
Parahippocampal and ambient gyri	0.012 (0.002)	0.025 (0.014)	0.012 (0.002)	0.026 (0.011)
Total brain	0.013 (0.002)	0.024 (0.009)	0.012 (0.001)	0.027 (0.012)
Caudate nucleus	0.019 (0.002)	0.041 (0.016)	0.018 (0.003)	0.043 (0.022)
Nucleus accumbens	0.019 (0.002)	0.037 (0.019)	0.018 (0.003)	0.050 (0.032)
Pallidum	0.022 (0.002)	0.050 (0.019)	0.021 (0.002)	0.063 (0.030)
Putamen	0.021 (0.003)	0.053 (0.013)	0.022 (0.003)	0.056 (0.025)
Striatum	0.020 (0.002)	0.047 (0.013)	0.020 (0.003)	0.049 (0.023)
Amygdala	0.011 (0.001)	0.023 (0.016)	0.011 (0.001)	0.018 (0.013)
Hippocampus	0.013 (0.001)	0.019 (0.015)	0.013 (0.002)	0.026 (0.018)
Thalamus	0.018 (0.002)	0.032 (0.016)	0.018 (0.002)	0.036 (0.019)
Hypothalamus	0.010 (0.002)	0.015 (0.017)	0.010 (0.002)	0.005 (0.040)

General acquisition parameters of the scans are described elsewhere [8]. Administration of ^18^F-fluorodeoxyglucose (^18^F-FDG) was performed during the clamp experiments as previously described [8]. All PET images were reconstructed using time-of-flight ordered subsets expectation maximization using 2 iterations and 28 subsets, including resolution recovery, and a 5 mm gaussian post-filter. The 10 min cardiac PET scan was reconstructed into 26 frames (1 × 10, 8 × 5, 4 × 10, 2 × 15, 3 × 20, 4 × 30, 6 × 60 s) and a 1 cm diameter volume of interest (VOI) was drawn in 10 consecutive slices in the ascending aorta for the creation of the initial phase of blood time activity curve. The dynamic brain PET images were reconstructed into frames of 5 min duration each. PET data were corrected for inter-frame motion. A probabilistic gray matter VOI template was applied using the PVElab software [24]. The blood time-activity curve was extended to the full duration of the brain PET measurement using a single exponential fit to activity concentrations measured in arterialized venous blood samples taken at set times during the scan. Regional net uptake rate of ^18^F-FDG (Ki) was expressed as plasma clearance, and it was calculated using a dual-phase basis function implementation of an irreversible two-tissue compartment model allowing for a change in rate constants during the glucose reduction phase for all investigated brain regions.

### Calculations and statistical analyses

Normal distribution for Ki values was verified with Shapiro-Wilk test, therefore parametric testing was used throughout. Differences between pre- and post-RYGB visits were assessed with paired t-tests. Due to the observational nature of the study and the small sample size no correction for multiple testing was performed. Correlation analyses were performed using Pearson’s test.

Effect size measurements were carried out with Hedges’ g formula and classified as follows: (a) “small” effect size for 0.2 < |g | <0.5; (b) “medium” effect size for 0.5 < |g | <0.8; (c) “large” effect size for |g | >0.8 [25].

Cluster analyses were carried out to understand how the brain regions cluster according to their changes in FDG clearance in response to RYGB. Hierarchical clustering with the Ward D2 method was performed to identify clusters of regions of interest (ROIs) with similar patterns in Ki changes. To determine the optimal number of clusters for the k-means algorithm, the average silhouette width was calculated across a range of cluster number (Supplementary Fig. 1).

Statistical analyses were performed and plots were created with R 4.2.3 and GraphPad Prism 9.

## Results

Full clinical outcomes following RYGB were published before [8], and demographic and anthropometrics of study participants are reported in supplementary table 1. All study participants were right-handed. Out of 8 female participants, 3 were nulliparas at the time of the investigation.

### Changes in regional brain glucose clearance after RYGB

Regional net uptake rates of ^18^F-FDG (Ki) for each analyzed region during normo- and hypoglycemia, before and after RYGB, are shown in Table 1. In absolute terms, the FDG uptake rate was highest in pallidum, putamen, and posterior cingulate gyrus, both during normo- and hypoglycemia, before and after surgery. The FDG uptake rate was lowest in the medial part of the anterior temporal lobe, the amygdala, and the hypothalamus, both during normo- and hypoglycemia, before and after surgery.

Before surgery, FDG uptake rate was significantly higher during hypoglycemia compared to normoglycemia in the whole brain (*p* = 0.003), and in most cortical and subcortical areas, but not in amygdala, hippocampus, and hypothalamus. After surgery, FDG uptake rate was higher during hypoglycemia compared to normoglycemia in all analyzed regions (*p* < 0.05), except for amygdala, hippocampus, and hypothalamus.

**Fig. 1:**
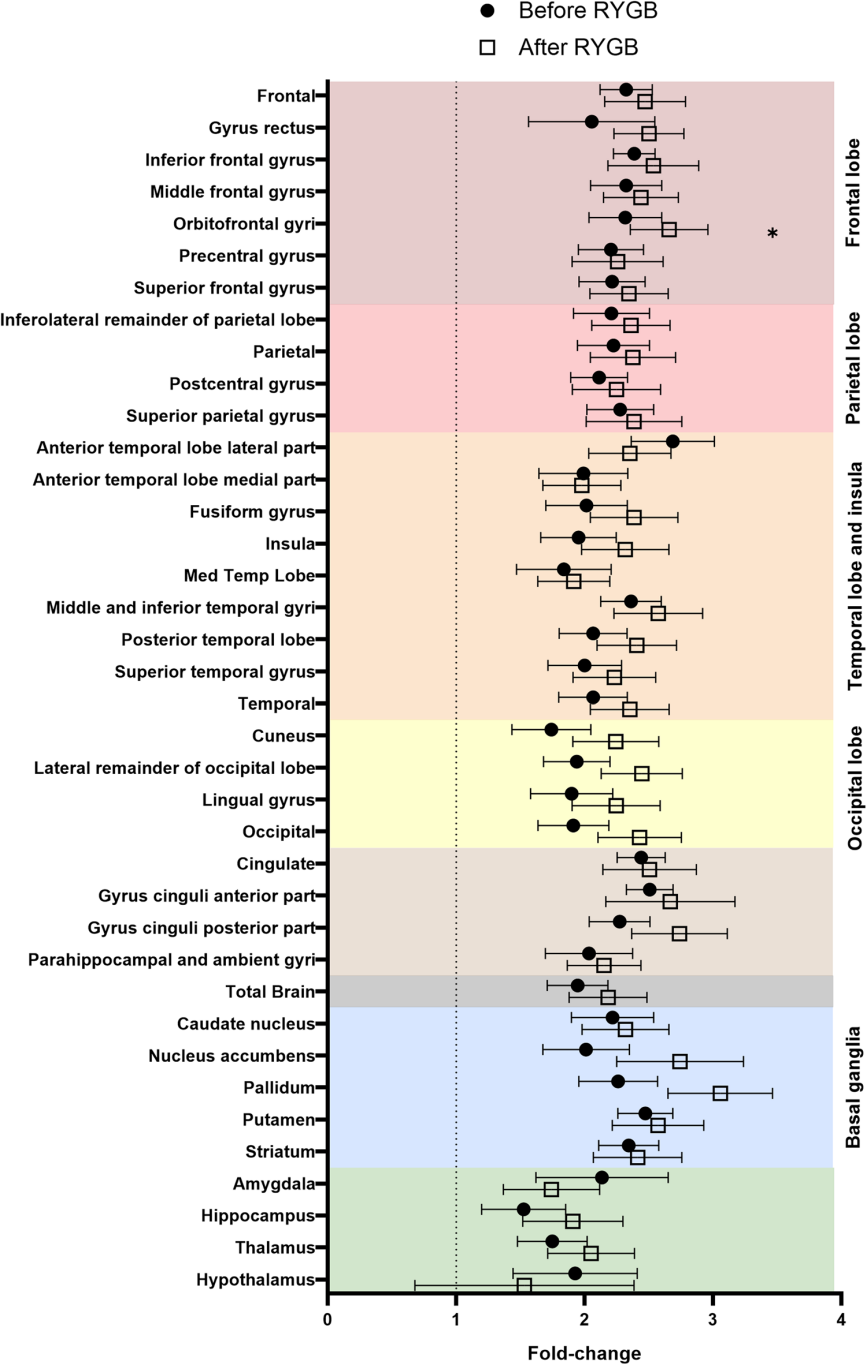
Fold-change (ratio) of regional FDG clearance during hypoglycemia vs normoglycemia before (filled circles) and after (open squares) RYGB. Paired t tests. *p < 0.05. Dots represent means and lines represent standard errors. Background colors represent the anatomical classification of the regions. Dark red: frontal cortex; bright red: parietal cortex; orange: temporal cortex; yellow: occipital cortex; brown: limbic cortex; black: whole brain; blue: basal ganglia; green: other subcortical nuclei

The fold-change in FDG uptake rate during hypoglycemia compared to normoglycemia was significantly increased after RYGB in the orbitofrontal gyri (*p* = 0.022) and tended to be higher in most cortical regions and the basal ganglia, but not in the anterior temporal lobe, amygdala, and hypothalamus, where it tended to be lower (Fig. 1). However, the difference in the fold-change among regions was not significant.

**Fig. 2:**
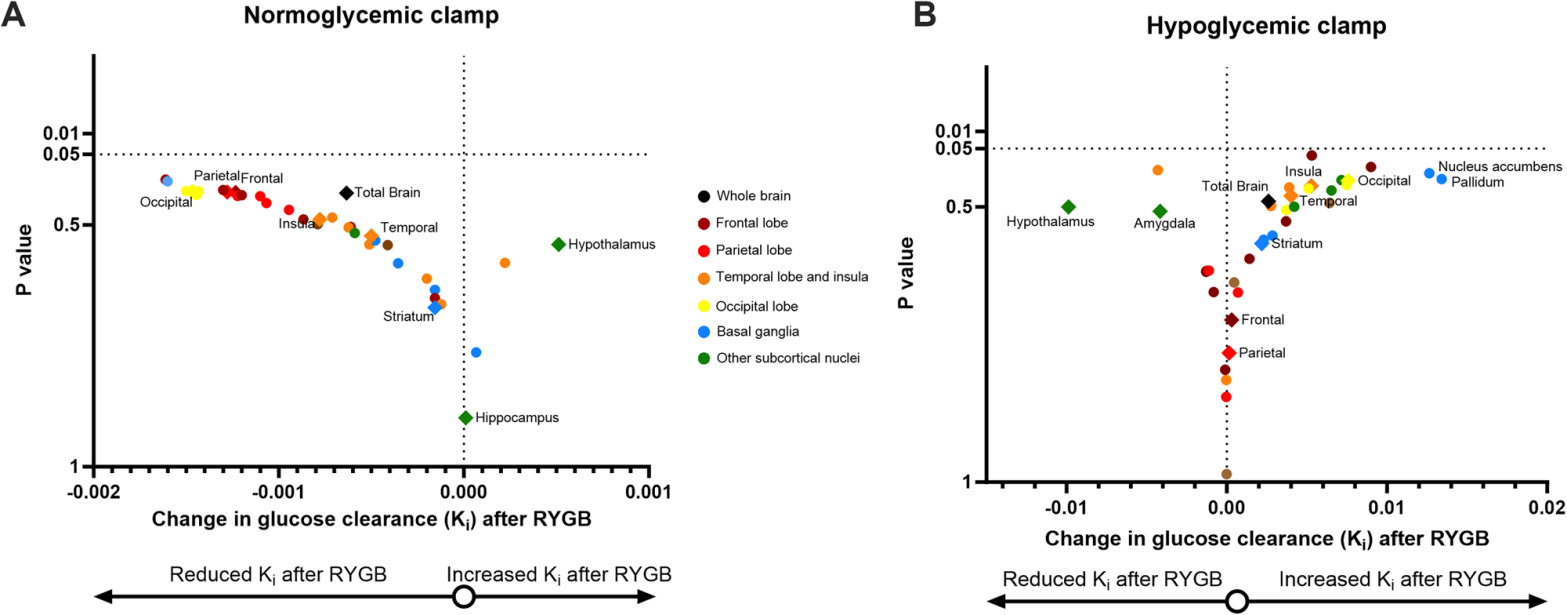
Volcano plots depicting changes in FDG clearance after RYGB. **A** Normoglycemic clamp. **B** Hypoglycemic clamp. X-axis: mean change of glucose clearance after RYGB. Y-axes: *p* values of paired t tests. Dots represent the single analyzed regions. The color coding relates to the anatomical localization of the region. Diamonds and name labels are given to the regions of main interest

Under experimental normoglycemia, FDG uptake rate tended to be lower after RYGB overall in the brain and in frontal, parietal and occipital areas, while it was almost unchanged in temporal regions and basal ganglia. In contrast, the hypothalamus tended to have a higher FDG uptake rate after RYGB (Fig. 2A). The Hedges’ g effect size of RYGB on the glucose clearance change of frontal, parietal and occipital cortex was “medium” (g > 0.5), yet higher than the effect on the whole brain (g = 0.35) (Supplementary Fig. 2A).

Under experimental hypoglycemia, FDG uptake rate tended to be higher in nucleus accumbens, pallidum, insula, and occipital cortex. No trends could be detected for either frontal or parietal regions, or for the striatum. Instead, hypothalamus and amygdala tended to have lower FDG uptake rate after RYGB (Fig. 2B). The effect size of RYGB on the glucose clearance change in the pallidum was “medium” (g = 0.52), while it was small or minimal in all other regions (Supplementary Fig. 2B).

### Post-RYGB changes of FDG clearance in functional brain networks

The effect sizes of regions functionally belonging to the default mode network, the behavioral control network, and the reward system were pooled together to get an average value. The effect of RYGB on FDG uptake rate of regions belonging to the behavioral control network was greater compared to the average effect on the brain and in the other two systems, both during normo- and hypoglycemia. During normoglycemia, the effect of RYGB on the behavioral control network was “large” (g = −1.03) (Table 2).

**Table 2:** Average effect size of RYGB on the FDG clearance in regions belonging to different brain functional networks. Values represent Hedges’ *g*

	Normoglycemia	Hypoglycemia
Reward system	−0.126	0.194
Behavioral control	−1.030	0.582
Default mode network	−0.576	0.220
Whole brain	−0.350	0.239

### Associations between changes in regional brain FDG clearance and clinical and neuroimaging variables

**Fig. 3:**
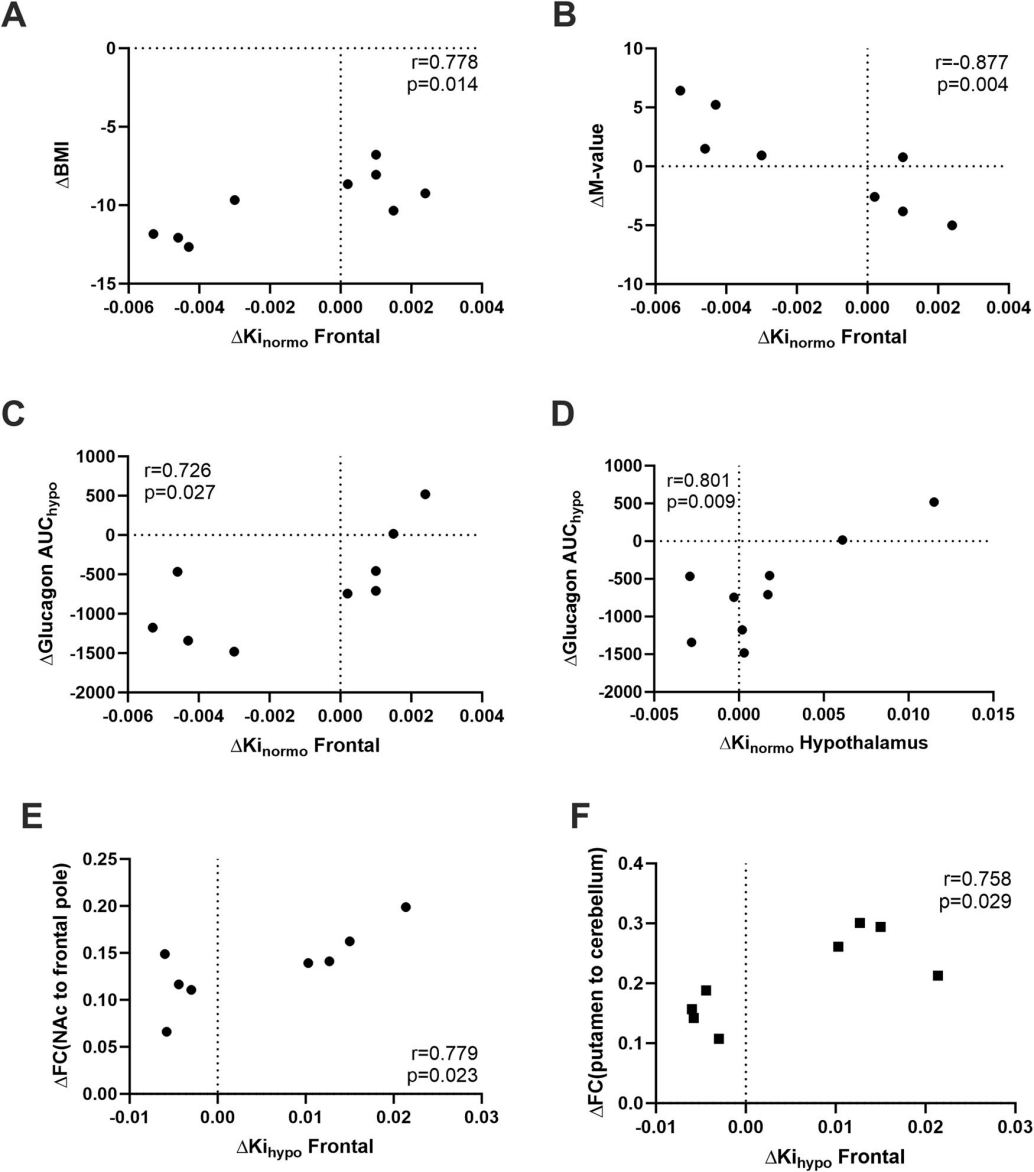
Correlation analyses between changes in FDG Ki after RYGB (X-axes) in the frontal cortex (**A**–**C, E,** **F**) and in the hypothalamus (**D**) and changes in other clinical and neuroimaging variables after RYGB. BMI body mass index, FC functional connectivity during the glucose-lowering phase of the clamp. NAc nucleus accumbens, AUC area under the curve

During experimental normoglycemia, there was a positive correlation between the BMI change after surgery and the post-surgical changes of FDG Ki in all analyzed regions (all *p* < 0.05), except for the hypothalamus (*p* = 0.31) and the hippocampus (*p* = 0.07) (Supplementary Table 2, Fig. 3A). Also, the post-surgical changes of the M-value was negatively correlated with the post-surgical changes of FDG Ki in frontal, parietal, temporal cortex, insula, striatum, and pallidum (all *p* < 0.05) (Supplementary Table 2, Fig. 3B). These results were confirmed in multiple univariate regression analyses, where the change in BMI and M-value acted as significant predictors of the glucose clearance change in most cortical and subcortical regions, but not in the hypothalamus (data not shown). Remarkably, post-surgery changes in normoglycemic FDG Ki of the frontal cortex and the hypothalamus correlated with the post-surgery change in glucagon secretion during the hypoglycemic clamp (*p* = 0.026 and *p* = 0.009, respectively) (Fig. 3C, D). We did not find any correlations between changes in regional FDG Ki and changes with the outcomes of the cognitive tests during hypoglycemia.

In a previously published paper, we showed increased functional connectivity after RYGB between the lateral hypothalamus and the hippocampus, as well as increased functional connectivity between the putamen and the cerebellum during the glucose-lowering phase of the clamp [9]. We found that these changes correlated with the change in FDG clearance in frontal, parietal, temporal, occipital cortex, insula, dorsal striatum, and the hippocampus (all *p* < 0.05, r > 0.700) (Fig. 3E, F).

### Cluster analyses

**Fig. 4:**
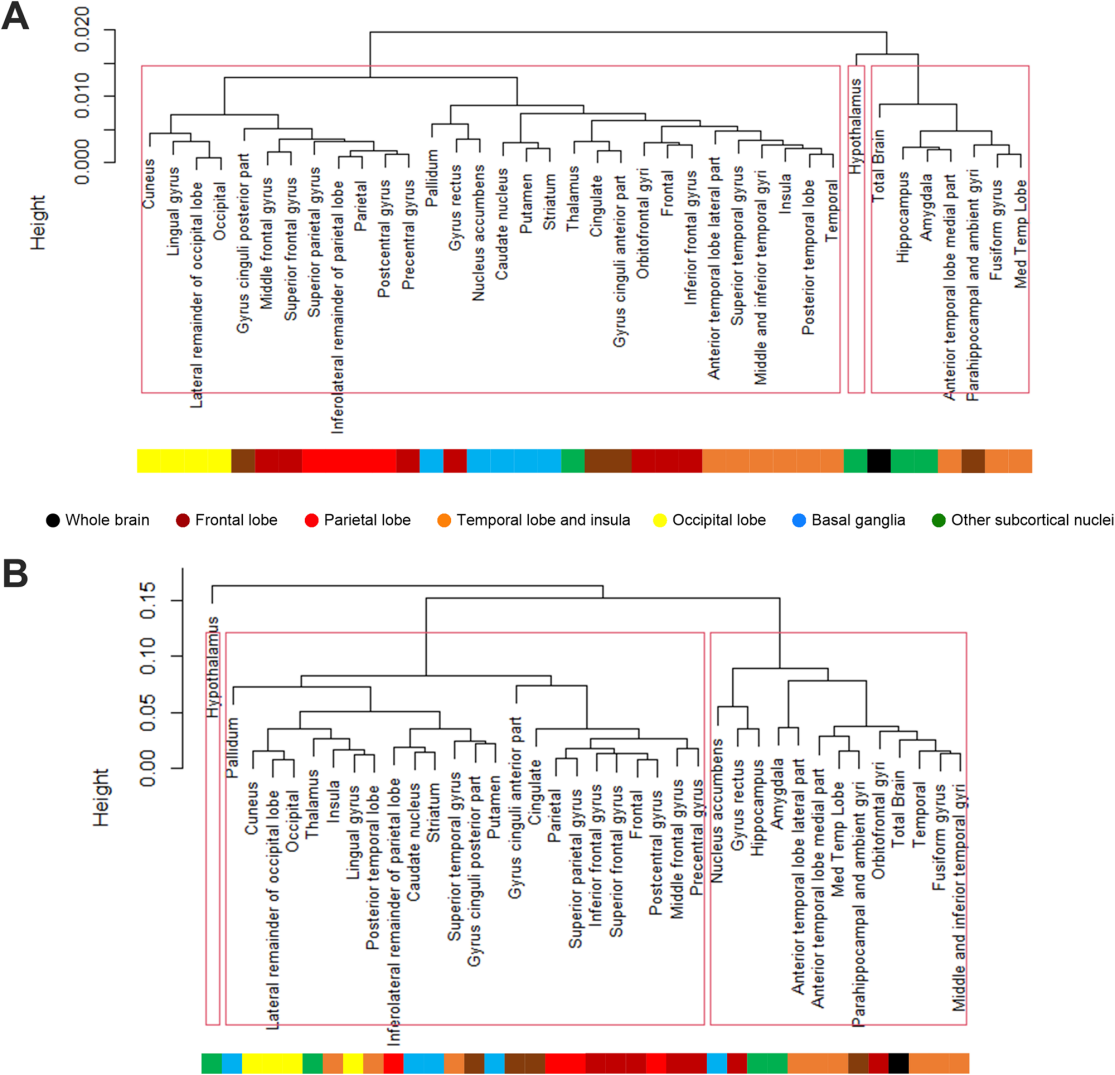
Hierarchical clustering of brain regions according to region similarity for changes in FDG clearance after RYGB. **A** Experimental normoglycemia. **B** Experimental hypoglycemia. Brackets represent hierarchical clustering and their height represents the distance among the clusters. The color coding represents the anatomical classification of the regions. Dark red: frontal cortex; bright red: parietal cortex; orange: temporal cortex; yellow: occipital cortex; brown: limbic cortex; black: whole brain; blue: basal ganglia; green: other subcortical nuclei

During experimental normoglycemia, three distinct regional clusters were identified according to their change in FDG clearance in response to RYGB. One cluster included the hypothalamus alone. A second cluster included amygdala, hippocampus, the parahippocampal cortex, and some temporal cortical regions. A third cluster included most cortical regions and the basal ganglia (Fig. 4A). During experimental hypoglycemia, three distinct clusters were identified as well. Again, the hypothalamus clustered alone. A second cluster included most regions of the temporal cortex, the hippocampus, the parahippocampal cortex, and the amygdala. A third cluster included the vast majority of cortical areas and the basal ganglia (Fig. 4B).

## Discussion

In this paper, we show how RYGB affects glucose turnover in 38 brain regions during experimental normo- and hypoglycemia. Our results support our hypothesis that RYGB differently affects various brain regions in terms of glucose metabolism, and that this possibly reflects how obesity surgery reshapes regional neural activity leading to favorable peripheral metabolic effects [4].

We assessed post-RYGB changes in regional glucose uptake rate as reflected by the relative amount of FDG extracted from blood, which is assumed to reflect glucose dynamics. FDG uptake rate during hypoglycemia was higher than during normoglycemia in all analyzed brain regions, both before and after RYGB. This was expected, since the brain needs to maintain an adequate influx of glucose to function properly, and therefore the relative glucose extraction is increased during hypoglycemia [26]. Interestingly, we show that after RYGB the hypo-to-normoglycemia ratio of FDG uptake rate tended to increase in almost all brain regions, but was lower instead in the anterior temporal lobe, amygdala, and hypothalamus. In fact, cluster analysis carried out according to regions’ similarity in the FDG uptake change after RYGB showed three distinct groups of brain regions, both in the settings of the normo- and the hypoglycemic clamp. Interestingly, the hypothalamus clustered alone, underpinning a different glucose handling compared to the rest of the brain.

Whole-brain glucose uptake during hyperinsulinemic normoglycemia is inversely correlated with insulin sensitivity [15]. Glucose uptake in the brain is considered to be largely mediated by astrocytes [27]. Therefore, astrogliosis, which is thought to reflect brain inflammation, could explain higher brain glucose uptake in insulin resistant individuals [15]. However, it is unlikely that brain inflammation occurs homogeneously throughout the brain, and our results support this hypothesis. In fact, obesity is specifically associated with hypothalamic neuroinflammation in animal data [28]. However, proof of obesity-associated hypothalamic neuroinflammation in humans is still lacking [28–30]. We rather show a trend for increased hypothalamic glucose uptake, which is in contrast with this hypothesis. Therefore, evidence about the relationship between obesity and brain inflammation are still contradictive and poorly understood.

To our knowledge, only three studies explored changes in regional brain glucose uptake after RYGB, although in different metabolic settings than in this study. In one study, glucose uptake was assessed during an OGTT [19]. Cortical regions, the hippocampus, and the putamen were analyzed, and all of them showed a decrease in glucose metabolic rate after RYGB, while the hypothalamus was not analyzed [19]. Hunt et al. analyzed glucose uptake during a mixed-meal test and showed that hypothalamic glucose uptake was exaggerated after RYGB, and this was interpreted as food ingestion being a greater physiological stimulus for the brain’s energy homeostasis network [22]. Tuulari et al. showed that RYGB and sleeve gastrectomy normalized brain glucose uptake in the main cortical areas and in the cerebellum during hyperinsulinemic normoglycemia [18], in line with our results. However, no data about glucose uptake in the hypothalamus were available. No previous study has evaluated regional brain glucose uptake during hypoglycemia.

In our study, RYGB tended to increase FDG uptake rate in the hypothalamus during experimental normoglycemia, but to reduce it during hypoglycemia, in contrast to most cortical regions and other subcortical nuclei. On the other hand, the effect of RYGB on glucose uptake was greatest when regions belonging to the behavioral control network were pooled together, both during normo- and hypoglycemia. Also, the hypo-to-normoglycemia ratio of FDG uptake rate was significantly increased in the orbitofrontal gyri, a key region in behavioral inhibitory control. This is consistent with Hunt et al., where restoration of normal inhibitory control responses to meal ingestion was shown after RYGB [22]. Also, this is in line with our previous finding of altered functional connectivity in inhibitory control networks in the glucose-lowering phase of the clamp in the same cohort of individuals [9].

We propose that after RYGB, glucose uptake during a hyperinsulinemic normoglycemic clamp is increased in glucose-sensing hypothalamic neurons. Therefore, they may sense a relatively higher systemic glucose availability compared to before surgery, leading to increased insulin secretion and lower levels of counterregulatory hormones, such as glucagon, catecholamines and cortisol [31]. This could contribute to lower glucose levels after RYGB, potentially including asymptomatic hypoglycemic episodes occurring in a significant proportion of patients [6]. During manifest hypoglycemia, glucose uptake in the hypothalamus might instead be decreased after RYGB, but increased in regions belonging to the inhibitory control network. However, a hyperinsulinemic clamp is not representative of a physiologic normoinsulinemic condition. Further, we did not find any correlations between changes in FDG uptake rate in the hypothalamus and changes in BMI and M-value. Altogether, glucose uptake in the hypothalamus might reflect glucose sensing, but other mechanisms are probably involved in its control of whole-body metabolism [10].

The clinical relevance of these findings is supported by the associations of post-RYGB changes in glucose uptake in cortical regions and the striatum during normoglycemia with BMI reduction and improved systemic insulin sensitivity. Interestingly, the post-RYGB change of hypothalamic and frontal cortex glucose uptake rate was associated with altered glucagon secretion during hypoglycemia after surgery. This suggests a link between brain glucose metabolism and the pancreatic islet’s control of glucose homeostasis, possibly via adaptation in the outflow of the autonomic nervous system and of pituitary-peripheral hormonal axes.

## Limitations

This study has several limitations. The sample size is small, so some analyses might be underpowered. Also, the spatial resolution of the FDG-PET is not optimal, therefore Ki assessments of smaller regions might be less accurate. The tracer signal during hypoglycemia was weaker than during normoglycemia since this part of the investigation was carried out longer after the administration of the radiolabeled ligand. This might have made the Ki assessments during the hypoglycemic phase less accurate.

No absolute calculations of brain glucose uptake during the hypoglycemic clamp could be made, since the brain’s lumped constant for FDG, which is necessary to convert the FDG uptake rate to absolute glucose uptake rates, most likely differs across varying glycemic levels [32]. Rather, animal studies showed higher lumped constant during hypoglycemia [33], meaning that the corresponding glucose uptake rate, even after adjustment FDG Ki for circulating glucose levels, would in reality be lower than during normoglycemia. Hyperinsulinemia was applied to achieve a constant stimulation of glucose uptake and to inhibit endogenous glucose production. This enabled a standardized assessment of insulin sensitivity and glucose turnover, but it does not represent a normal physiological state. Finally, this study included only participants without diabetes, and future work is warranted to also address patients with type 2 diabetes.

## Conclusions

This exploratory study suggests that RYGB has a heterogenous effect on brain glucose metabolism at a regional level. After RYGB, there is a reduction in the brain's clearance of plasma glucose during normoglycemia, but an increase during hypoglycemia. This was mostly evident in regions belonging to the behavioral control network. However, these trends were opposite in the hypothalamus. RYGB-induced changes in regional glucose uptake rate assessed during hyperinsulinemic normoglycemia were also correlated with weight loss and improvement of systemic insulin sensitivity. These results show the feasibility to study regional brain glucose turnover with dynamic FDG-PET assessment at different blood glucose levels. They highlight the brain effects of RYGB, suggesting behavioral and neuroendocrine adaptations that potentially contribute to its antidiabetic effects. This pilot study should be followed by additional work in larger cohorts to further define the brain effects of RYGB and the mechanisms involved in crosstalk between brain regions and with peripheral tissues.

## Supplementary information

supplementary material

## Data Availability

Data are available upon request to the corresponding author.
