# Supplementary material for: Regional brain glucose uptake following gastric bypass surgery during normo- and hypoglycemic clamp: a pilot FDG-PET study
Source: Endocrine. 2024 Dec 7;88(1):110–21. doi: 10.1007/s12020-024-04127-1 (PMC11933149; doi:10.1007/s12020-024-04127-1)
Supplement: Supplementary file 1 — supplementary material [file 12020_2024_4127_MOESM1_ESM.docx]

**Regional brain glucose uptake following gastric bypass surgery during normo- and hypoglycemic clamp: A pilot FDG-PET study**

Giovanni Fanni, Sofia Kvernby, Sadiq Radhi, Argyri Mathioudaki, Magnus Sundbom, Sven Haller, Erika Roman, Johan Wikström, Mark Lubberink, Jan W Eriksson^1^

*Endocrine*

^1^Department of Medical Sciences, Clinical Diabetes and Metabolism, Uppsala University, Uppsala, Sweden. Mail address: [jan.eriksson@medsci.uu.se](mailto:jan.eriksson@medsci.uu.se)

**Supplementary material**

**Supplementary Table 1**

Demographics and weight characteristics of the study participants. Data are presented as mean (SD) or median (IQR) as appropriate.

|  | Before RYGB | After RYGB | P-value |
| --- | --- | --- | --- |
| Sex (M/F) | 3/8 |  |  |
| Age (years) | 35 (8) |  |  |
| Weight (kg) | 113.8 (109.7-132.5) | 83.3 (77.2-98.5) | 0.003 |
| BMI (kg/m^2^) | 40.2 (3.6) | 29.9 (4.0) | <0.001 |

**Supplementary Table 2**. Correlation analysis between changes in regional glucose uptake rate (K_i_) after RYGB during normoglycemia and changes in body mass index (BMI) and M-value after RYGB. Pearson’s correlations.

|  | Change in BMI | | Change in M-value | |
| --- | --- | --- | --- | --- |
| Change in Ki after RYGB | R | P | R | P |
| Frontal | 0.778 | 0.014 | -0.877 | 0.004 |
| Gyrus rectus | 0.833 | 0.005 | -0.897 | 0.003 |
| Inferior frontal gyrus | 0.874 | 0.002 | -0.824 | 0.012 |
| Middle frontal gyrus | 0.818 | 0.007 | -0.771 | 0.025 |
| Orbitofrontal gyri | 0.801 | 0.010 | -0.905 | 0.002 |
| Precentral gyrus | 0.828 | 0.006 | -0.760 | 0.029 |
| Superior frontal gyrus | 0.837 | 0.005 | -0.738 | 0.037 |
| Inferolateral remainder of parietal lobe | 0.847 | 0.004 | -0.789 | 0.020 |
| Parietal | 0.821 | 0.007 | -0.755 | 0.030 |
| Postcentral gyrus | 0.795 | 0.010 | -0.774 | 0.024 |
| Superior parietal gyrus | 0.782 | 0.013 | -0.643 | 0.086 |
| Anterior temporal lobe lateral part | 0.754 | 0.019 | -0.743 | 0.035 |
| Anterior temporal lobe medial part | 0.674 | 0.046 | -0.354 | 0.390 |
| Fusiform gyrus | 0.825 | 0.006 | -0.515 | 0.192 |
| Insula | 0.866 | 0.003 | -0.748 | 0.033 |
| Med temp lobe | 0.764 | 0.017 | -0.452 | 0.260 |
| Middle and inferior temporal gyri | 0.801 | 0.009 | -0.715 | 0.046 |
| Posterior temporal lobe | 0.825 | 0.006 | -0.785 | 0.021 |
| Superior temporal gyrus | 0.782 | 0.013 | -0.857 | 0.007 |
| Temporal | 0.847 | 0.004 | -0.754 | 0.031 |
| Cuneus | 0.882 | 0.002 | -0.599 | 0.116 |
| Lateral remainder of occipital lobe | 0.856 | 0.003 | -0.660 | 0.075 |
| Lingual gyrus | 0.859 | 0.003 | -0.731 | 0.039 |
| Occipital | 0.882 | 0.002 | -0.678 | 0.064 |
| Cingulate | 0.876 | 0.002 | -0.707 | 0.050 |
| Anterior part of cingulate gyrus | 0.833 | 0.005 | -0.618 | 0.103 |
| Posterior part of cingulate gyrus | 0.878 | 0.002 | -0.767 | 0.026 |
| Parahippocampal and ambient gyri | 0.821 | 0.007 | -0.607 | 0.110 |
| Total brain | 0.396 | 0.291 | -0.394 | 0.334 |
| Caudate nucleus | 0.841 | 0.004 | -0.846 | 0.008 |
| Nucleus accumbens | 0.910 | 0.001 | -0.690 | 0.058 |
| Pallidum | 0.925 | 0.000 | -0.774 | 0.024 |
| Putamen | 0.934 | 0.000 | -0.812 | 0.014 |
| Striatum | 0.894 | 0.001 | -0.850 | 0.008 |
| Amygdala | 0.776 | 0.014 | -0.552 | 0.156 |
| Hippocampus | 0.629 | 0.069 | -0.263 | 0.529 |
| Thalamus | 0.902 | 0.001 | -0.695 | 0.056 |
| Hypothalamus | 0.377 | 0.317 | -0.646 | 0.083 |

**Supplementary Figure 1**. Cluster analyses. Silhouette width plot to identify the optimal number of clusters. (A) Experimental normoglycemia. (B) Experimental hypoglycemia.

** Supplementary Figure 2**. Bar plots depicting the effect size of RYGB on the regional glucose clearance change. X-axis: Hedges’ g value. (A) Normoglycemic clamp. (B) Hypoglycemic clamp.
